# Martin Baethge Obituary

**DOI:** 10.1186/s12651-018-0236-x

**Published:** 2018-03-01

**Authors:** Susan Seeber

**Affiliations:** Georg-August-Universitat Gottingen, Göttingen, Germany

Martin Baethge, Professor of Sociology at the University of Göttingen and until recently an active member of the editorial committee, passed away quite unexpectedly after a short illness on 4 January 2018 at the age of 78.

As a researcher and teacher, Martin Baethge significantly shaped the profile of sociology at Göttingen for almost five decades. He studied sociology, pedagogy, philosophy, and politics at the University of Göttingen and the Free University of Berlin. With his dissertation ‘Economic interests and education policy. A contribution to economic and educational sociology concerning the relationship of economic interests and education policy, as illustrated by the example of the education policy-related activities of enterpreneurial umbrella organisations’, Martin Baethge came to the University of Göttingen during the heated disputes of the 1960s; in 1969, he was awarded his doctorate for this publication in Hanover. The title of his dissertation alone makes it clear that it paved the way for Martin Baethge’s future research: He was always concerned with the perspectives of working people, democratisation processes, shaping and steering policies, organised work that shapes occupations, and vocational training. He was always scrutinising working conditions, criticising claims to power and the hegemony of economically powerful players, and demanded participation opportunities for everyone. The focus of his attention were the ‘losers’ of social modernisation processes; young people and adults who had difficulty accessing socially valuable commodities, such as finding vocational training, further education, and gainful employment. In 1973, he was appointed as professor of sociology at the University of Göttingen, a post he held until 2004.

Martin Baethge confronted the world of work and vocational training in many different publications. He criticised the persistence of social institutions in the structures and regulation mechanisms of a classically industrial society; he called for reforms, mainly with respect to vocational training based on a traditional industrial model; and he urged people to challenge their positions. His work was always shaped by conceptual clarity, analytic astuteness, and pointed messages. One of Martin Baethge’s works from 1978, ‘Training problems for young people and problems young people have starting’, was a topic that was to shape his sociological research for years to come. In the last two decades, these works were firmly anchored in national educational reporting, where he made his voice heard regarding issues concerning unequal opportunities when transitioning to training; he also called for missing system perspectives in the transition structures, and while doing so, he did not shy away from political confrontation. Martin Baethge contributed to educational reporting from the outset, helping to shape both the concept and the content. His final works focussed increasingly on growing integration problems within vocational training, problems concerning the professional integration of immigrants, the shift of co-ordinates in the vocational training system, and the associated new challenges in steering vocational training. He was always an active, stimulating, and politically-minded partner, both here and in related projects that dealt with outcome and steering issues within vocational training.

Apart from vocational training issues—the general common theme of his works—Martin Baethge mainly addressed the radical changes to industrial work caused by technological reforms and linked his analyses on operational rationalisation processes to the demand for social reform prospects, which always included a more social working world. For him, science was also always connected to discovering problem areas and pointing out action perspectives. Publications such as ‘The Future of Employees’ (1986) and ‘Goodbye to Industrialism’ (2006) were widely recognised beyond sociology. Looking at digitalisation processes in society and the working world, he developed new ideas for researching and shaping service work in a world of digital change with unwavering energy, even in the last few months of his work.

In parallel to these heavily theoretical issues, he also tried to introduce solutions to political and practical problems, where he also addressed key international issues. Here is a detailed example that shows how he was able to initiate and implement extensive research activities in a targeted manner: From 2002, he worked on a project with the Department of Business Education at the University of Göttingen for the purposes of carrying out an international assessment in the field of vocational education and training. The objective was to integrate sociological and politological categories with those of curriculum, instruction, and assessment research. This work, initially commissioned by the Federal Ministry of Economics, resulted in large-scale international collaboration. From an international perspective (16 developed countries from three continents and the CEDEFOP), it was also important that the key objective of vocational training was the individual political and social support for young people and young adults and not just the view that they should ‘get in shape’ for one specific job—which is an internationally widespread view. The fact that vocational training must also secure a qualified workforce for a state was never contested by Martin Baethge. The programme he developed appeared in book format in 2006 (‘PISA-VET: A Feasibility Study’). The proposal was so well-received that further preparatory studies followed. Martin Baethge’s creative force became obvious: 359 vocational training experts in eight European countries contributed to creating requirements for developing professional assessments (‘Feasibility Study VET-LSA’ 2009). All of this resulted in the ASCOT programme, launched by the Federal Ministry for Education and Research, through which 21 research projects in the areas of technology, economics, health, and care explored the bases of professional skills development. Vocational training research owes Martin Baethge a great debt of gratitude for having so successfully carried out these activities, which are waiting to be continued both nationally and internationally. This initiative is further proof of the impact his research activities have had beyond sociology.

Martin Baethge was very well-connected with the University of Göttingen and the town itself. Deeply rooted in his home town Göttingen he declined calls to the Technical University of Berlin and the Bielefeld University. As early as 1968, Martin Baethge co-founded the Institute of Sociology Research (Soziologisches Forschungsinstitut, SOFI) in Göttingen. Later on, he became its director, and from 2006 until his unexpected death, he was President of the Institute—a position that he filled actively and with undiminished creativity to the end.

Martin Baethge was a co-editor for the Journal for Labour Market Research from 2008, which at the time was known under the German title ‘Zeitschrift für ArbeitsmarktForschung’. He often took part and engaged in editorial meetings and brought his high level of professional expertise to the field of vocational training research—also as the co-editor and author of special issues.

With the passing of Martin Baethge, the world of educational, vocational, and working sociological research has lost an outstanding researcher, a key driving force, and a valiant colleague, who did not shy away from conflict. Many colleagues have not only lost a dedicated, creative, and intellectually stimulating research partner who always assumed responsibility for the common good, but, also a charming and generous friend who was a good listener—and not just over the odd glass of good wine—and scrutinised, gave advice, and provided support wherever he could. The great appreciation for his work and immense gratitude for Martin Baethge will linger on.

